# Intrinsic and Dopant-Related Luminescence of Undoped and Tb Plus Tm Double-Doped Lithium Magnesium Phosphate (LiMgPO_4_, LMP) Crystals

**DOI:** 10.3390/ma13092032

**Published:** 2020-04-27

**Authors:** Wojciech Gieszczyk, Paweł Bilski, Anna Mrozik, Mariusz Kłosowski, Barbara Marczewska, Anna Sas-Bieniarz, Marcin Perzanowski, Tetiana Zorenko, Yuriy Zorenko

**Affiliations:** 1 Institute of Nuclear Physics, Polish Academy of Sciences, Radzikowskiego 152, PL31342 Krakow, Poland; pawel.bilski@ifj.edu.pl (P.B.); anna.mrozik@ifj.edu.pl (A.M.); mariusz.klosowski@ifj.edu.pl (M.K.); barbara.marczewska@ifj.edu.pl (B.M.); anna.bieniarz@ifj.edu.pl (A.S.-B.); marcin.perzanowski@ifj.edu.pl (M.P.); 2 Institute of Physics, Kazimierz Wielki University in Bydgoszcz, Powstancow Wielkopolskich 2, PL85090 Bydgoszcz, Poland; tetiana.zorenko@ukw.edu.pl (T.Z.); zorenko@ukw.edu.pl (Y.Z.)

**Keywords:** lithium magnesium phosphate, crystal growth, stimulated luminescence phenomena, defects, rare-earths, temperature dependence

## Abstract

In this work, the luminescence properties of undoped, Tm^3+^ doped, and Tb^3+^ plus Tm^3+^ double-doped crystals of the lithium magnesium phosphate (LiMgPO_4_, LMP) compound were investigated. The crystals under study were grown from melt using the micro-pulling-down method. The intrinsic and dopant-related luminescence of these crystals were studied using cathodo-, radio-, photo-, and thermoluminescence methods. Double doping with Tb^3+^ and Tm^3+^ ions was analyzed as these dopants are expected to exhibit an opposite trapping nature, namely to create the hole and electron-trapping sites, respectively. The spectra measured for the undoped samples revealed three prominent broad emission bands with maxima at around 3.50, 2.48, and 1.95 eV, which were associated with intrinsic structural defects within the studied compound. These were expected due to the anion vacancies forming F^+^-like centers while trapping the electrons. The spectra measured for Tb and Tm double-doped crystals showed characteristic peaks corresponding to the 4f–4f transitions of these dopants. A simplified model of a recombination mechanism was proposed to explain the temperature dependence of the measured thermally stimulated luminescence spectra. It seems that at low temperatures (below 300 °C), the charge carriers were released from ^5^D_3_-related Tb^3+^ trapping sites and recombination took place at Tm-related sites, giving rise to the characteristic emission of Tm^3+^ ions. At higher temperatures, above 300 °C, the electrons occupying the Tm^3+^-related trapping sites started to be released, and recombination took place at ^5^D_4_-related Tb^3+^ recombination centers, giving rise to the characteristic emission of Tb^3+^ ions. The model explains the temperature dependence observed for the luminescence emission from double-doped LiMgPO_4_ crystals and may be fully applicable for the consideration of emissions of other double-doped compounds.

## 1. Introduction

Stimulated luminescence phenomena are extensively exploited as great research tools in the basic research of materials [1,2]. As these phenomena are extremely sensitive to defects in solids, they can, therefore, be utilized to study these defects. The basic physical effect leading to the production of stimulated luminescence is the trapping of charge carriers, i.e., the electrons and holes pairs produced during the exposure of the material to an external source of energy can be captured at defect sites in the material. In general, defect sites in solids can be divided into two categories: (1) those naturally present in the material (also called intrinsic defects) and (2) those produced by external means, such as intentionally doping the sample with, e.g., rare-earth (RE) elements. A well-known example of the first category is a negative ion vacancy creating the so-called F-center when trapping the electron. An example of the second category is a lattice vacancy caused by an impurity ion of a higher valence located at the position of a lattice ion (e.g., a trivalent cation impurity in a divalent lattice). This type of defect will induce the formation of a cation vacancy in the lattice to maintain charge neutrality. In turn, cationic vacancies are potential sites for trapping holes. It should be noted here that many other types of defects exist in solids that can act as electron or hole trapping centers. Revealing the nature of those defects constitutes a major part of research on luminescent materials [3]. The most commonly used for this purpose is the thermoluminescence (TL) phenomenon, but luminescence triggered by optical stimulation (optically stimulated luminescence, OSL) has become a more and more widely applied measurement technique. Among other methods, probably the most popular in materials research are photo-, radio-, and cathodoluminescence methods (PL, RL, and CL, respectively). TL is commonly known as a technique used in ionizing radiation dosimetry and dating, but it also helps in the determination of luminescence mechanisms, trapping parameters, energy levels of defects in solid, etc. In other words, one can say that TL, together with the other abovementioned stimulated luminescence techniques, constitutes a complementary method in the field of materials research [4,5].

Stimulated luminescence techniques are widely used for materials screening. This is especially related to searching for new materials whose luminescent properties would satisfy the needs of many different fields. Nowadays, the RE-doped materials play an important role, especially in modern technologies, e.g., for car catalyst production (La, Ce, Pr, Nd), solid magnets (Pr, Nd, Tb, Dy), optical filters (La, Ce, Pr, Nd), and phosphors (Y, La, Ce, Eu, Gd, Tb, Tm). This last case is recently the most extensively studied regarding the possibility of the production of white-light-emitting diodes, but also for high-sensitive radiation dosimetry, whose requirements are still increasing due to the ever-increasing number of applications of ionizing radiation.

Among materials that have recently been considered as good candidates for application in radiation dosimetry, the lithium magnesium phosphate (LiMgPO_4_, LMP) compound seems the most promising. Although the olivine-type compound’s crystal structure has been investigated since 1982 [6], the first research report on the luminescence characteristics of this material is probably dated to 2010 [7]. Since then, a large number of scientific papers devoted to luminescence investigations of this material have been published [8,9,10,11,12,13]. In most cases, samples in the form of powder [9], cold-pressed and sintered pellets [8], or fluoropolymer foils [13] have been studied. Recently, this material was also successfully crystallized from melt using a novel micro-pulling-down (MPD) crystal growth technique [14,15,16,17,18]. Further, the melt-grown crystals were found to show substantial advantages over samples in the form of powders [19], which were manifested as significantly reduced luminescence at the low-temperature range that may strongly affect the fading properties of this material.

Because the luminescence is extremely sensitive to defects in solids, investigations on the intrinsic and dopant-related defects should constitute the major part of work on the development of new dosimetric materials. However, in the case of the LMP compound, the works on intrinsic defect-related luminescence are practically unavailable, and most of the papers are focused on RE dopant-related phenomena. Although the luminescent properties of this relatively new and promising phosphor have been of interest to several research groups for almost a decade, there is still only very limited knowledge on the luminescence mechanisms, especially in the case of nominally pure samples of the material. There are probably only two research papers available that are devoted to the intrinsic-defect-related luminescence in LMP cold-pressed and sintered pellets [20,21]. However, in the case of crystals, the situation may look slightly different. As reported many times [14,15,16,17,18,19], high-temperature crystallization changes the energetic distribution of TL-related electron traps in materials, which are strongly manifested in the observed differences between the glow-curves measured for materials in different forms (namely powders and crystals). This may be related to the creation of many different types of structural defects. It is known that the main type of defects in oxide materials are the anion vacancies, creating the F^+^ and F centers when trapping one and two electrons, respectively, and their aggregates, which are commonly considered to be luminescence-related centers in materials. Apart from the anion vacancies, other types of defects should also be taken into account, e.g., antisite defects (ADs), which are often created during high-temperature crystal growth experiments.

In this work, the luminescence spectra of the undoped and double-doped LMP crystals were investigated. The spectra of the nominally pure samples were expected to show only intrinsic defect-related luminescence, and possibly, some signals originating from the trace impurities of raw materials, while the spectra of double-doped samples may be attributed to both intrinsic and dopant-related defect luminescence. Because of the excellent chemical stability, charge stabilization, and simplicity of synthesis, LiMgPO_4_ constitutes a suitable host lattice for different doping ions. In this work, Tb^3+^ and Tm^3+^ ions were chosen as co-dopants because they were expected to show an opposite trapping nature, namely to create the hole and electron trapping sites, respectively [22]. The measurements were performed using TL, CL, PL, and RL methods, and the observed emission bands were interpreted based on the available literature data. It is worth mentioning that the TL method is commonly used for investigations of the so-called energy-storage phosphors, e.g., for the routine dosimetric applications, while the RL and CL methods imitate the scintillation phenomenon well, where the stage of trapping and storing the free charge carriers is usually not considered.

## 2. Materials and Methods

### 2.1. Materials Preparation

A nominally pure lithium magnesium phosphate compound was prepared using the conventional solid-state synthesis in air. Monohydrate lithium hydroxide (LiOH·H_2_O, AlfaAesar, Haverhill, MA, USA; purity 99.995%), hexahydrate magnesium nitrate (Mg(NO_3_)_2_·6H_2_O, AlfaAesar, Haverhill, MA, USA; purity 99.999%), and ammonium dihydrogen phosphate (NH_4_H_2_PO_4_, Avantor Performance Materials, Gliwice, Poland; purity 99.5%) were used as the initial reagents. The appropriate amounts of these reagents were thoroughly mixed before the thermal treatment, including several cycles at the temperatures ranging from 200 to 750 °C. The final product was then grounded and sieved to achieve a grain size below 212 µm. In the case of the double-doped compound, Tb_4_O_7_ (AlfaAesar Haverhill, Massachusetts, USA; purity 99.9%) and Tm_2_O_3_ (Sigma-Aldrich, Saint Louis, MO, USA; purity 99.9%) oxides were utilized. The dopant concentrations were 0.2 and 0.6 mol% for Tb and Tm ions, respectively. The more detailed description of LMP powder preparation using the solid-state reaction can be found elsewhere [17,18,20].

It should be mentioned here that our lithium magnesium phosphate powders were synthesized in a one-step reaction where only gas products of water, ammonia, and nitrogen dioxide were emitted outside the crucible. The final product of the solid-state reaction (LiMgPO_4_) remained in the crucible, and based on the weight of the product, we estimated that the synthesis yield was almost 98%–100%.

### 2.2. Crystal Growth Experiments

**Figure 1:**
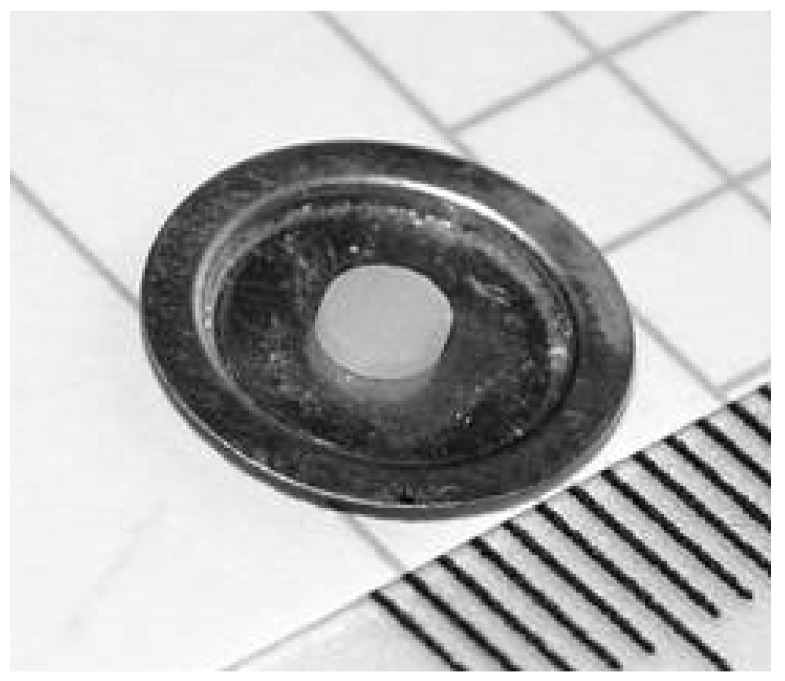
An exemplary LiMgPO_4_ (LMP) crystal grown from melt using the micro-pulling-down method. The crystal was placed on a stainless steel cup that held the sample during luminescence measurements inside the Risø TL/OSL DA-20 reader.

The LMP crystals were grown from melt using the MPD method at the Institute of Nuclear Physics at the Polish Academy of Sciences (IFJ PAN) in Krakow, Poland, by utilizing the unique crystal growth device manufactured by Cybyerstar, France. An inductive furnace powered by a 20 kW radio-frequency generator, typically operating at a 12.7 kHz frequency, constituted the main part of the system. The as-prepared powders (fractions of around 1 g) were used as initial materials in the crystal growth experiments. The crystals were grown using the following heating profile: (I) heating of the system to the LMP melting temperature (1025 °C/0.5 h), (II) keeping a constant temperature during the crystal growth, and (III) cooling the system down to room temperature (RT/0.5 h). The crystals were grown in the graphite crucible with an additional molybdenum overlay, which significantly improved the heat generation properties. To protect all the applied thermal setup elements from the oxidation, the crystals were grown in a protective inert gas atmosphere (Ar). The applied pulling rate was 0.2 mm/min. It is also worth mentioning here that relatively high pulling rates, applicable in the MPD method, in combination with the small amounts of required starting materials, make the MPD method a highly-efficient tool for materials engineering. The obtained rod-shaped crystal of around 3 mm in diameter and several centimeters in length were then cut into slices of 1 mm in thickness. An exemplary crystal slice is shown in Figure 1. To minimize the differences in the signal intensities resulting from the different sizes of crystals, all investigated samples were weighed and the signal intensity measured for a given sample was normalized to its mass.

### 2.3. XRD Phase Analysis

The crystal structure of the samples was studied using X-ray diffraction (XRD). The diffraction measurements were carried out using a PANalytical X’Pert Pro diffractometer (Malvern Panalytical B.V., Almelo, The Netherlands) using the Cu *K*_α1_ line (wavelength λ = 0.154 nm), operating at 40 kV and 30 mA. The experiments were performed in θ/2θ Bragg-Brentano geometry in a 2θ range from 15° to 75° with an instrumental step of 0.05°. The patterns were corrected for the instrumental background, and the contribution of the Cu *K*_α__2_ line was subtracted using the Rachinger algorithm. Before the measurements, the samples were powdered with an agate mortar.

### 2.4. Luminescence Measurements

The luminescence emission spectra of the studied crystals were measured under three different modes, namely via thermal stimulation, 10 keV electron beam excitation, and ^90^Sr/^90^Y beta ray excitation, corresponding to the TL, CL and RL phenomena, respectively. The TL emission spectra were measured at the IFJ PAN in Krakow, Poland, using the semi-automatic Risø DA-20 reader (Risø DTU, Roskilde, Denmark). The reader was nominally equipped with a bialkali photomultiplier tube (EMI 9235QB) and two sources of ionizing radiation, namely ^241^Am alpha particles and ^90^Sr/^90^Y beta radiation sources. The detailed specification of the reader and its performance can be found in Bilski et al. [23]. It should be mentioned that for TL emission spectra measurements, a standard photomultiplier tube was replaced by the Ocean Optics QEpro 00689 grating spectrometer (Ocean Optics B.V., Ostfildern, Germany) connected to the reader using a 400 µm optical fiber. This spectrometer allowed for measuring emission spectra over the spectral range from 200 to 1000 nm with a 4 nm resolution. Thus, the Risø reader was used for sample irradiation and thermal stimulation, but the signal registration was realized by the spectrometer. The time–temperature profile was set from RT to 500 °C with a constant heating rate of 2 °C/s. The samples were heated in a protective inert gas atmosphere (Ar). The volumetric flow rate of Ar was around 0.1 L/min. The preliminary processing of the measured glow-curves and emission spectra was conducted using dedicated GlowVIEW computer software [24].

The RL measurements were performed at IFJ PAN, Krakow, using an external beta-irradiator equipped with a ^90^Sr/^90^Y beta source. The dose rate of the source was estimated to be around 120 mGy/s at the sample position. The 400 µm diameter optical fiber with the LiMgPO_4_ crystal fixed at the end was placed directly under the radiation source and the opposite end of the fiber was connected to the spectrometer. The RL spectra were registered under the same conditions as TL spectra, namely from 200 to 1000 nm with a 4 nm resolution. The RL spectra were recorded at RT.

The CL and PL spectra measurements were performed at the Institute of Physics, Kazimierz Wielki University (UKW), in Bydgoszcz, Poland. The CL spectra were measured at RT using a Stellar Net grating spectrometer (StellarNet Inc, Tampa, FL, USA), which allowed for measuring the emission spectra over the spectral range from 200 to 1200 nm with a 1 nm resolution. For the sample excitations, the 10 keV electron beam was delivered from the electron gun of a JEOL SEM microscope (HEOL, Tokyo, Japan). Because this work was devoted to the investigation of the intrinsic defect-related luminescence, it is important to note that the 10 keV electrons delivered for CL spectra measurements were not able to create new types of defects, e.g., oxygen vacancies and antisite defects, due to the high displacement energy required for the knock-out mechanism in binary and complex compounds [25]. The PL excitation spectra and decay kinetics were recorded on an Edinburgh FS 5 spectrofluorometer (Edinburgh Instruments, Edinburg, UK) using a 150 W Xe lamp as the excitation source. It is worth mentioning that the RL and CL methods imitate the scintillation phenomenon well, where the stage of trapping and storing the free charge carriers is usually not considered.

## 3. Results and Discussion

### 3.1. XRD Phase Analysis

**Figure 2:**
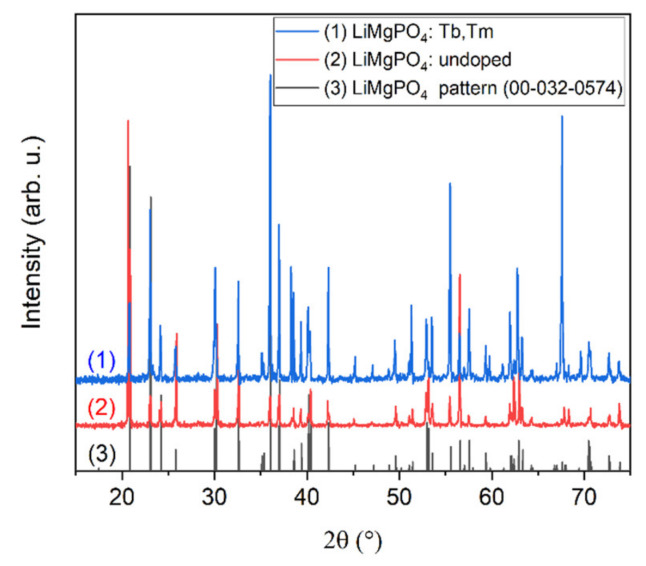
The XRD diffraction patterns of the undoped and Tb^3+^ plus Tm^3+^ double-doped LMP crystals investigated in this work. The patterns were shifted vertically for more clarity.

The crystal structure of the undoped and Tb^3+^ and Tm^3+^ double-doped crystals was checked using the XRD technique. Representative XRD diffraction patterns from the LMP crystals are shown in Figure 2. The positions of the XRD peaks were in agreement with the reference pattern taken from the International Center for Diffraction Data database (No. 00-032-0574), confirming that the samples were single-phase. The materials had an orthorhombic crystal structure with *Pnmb* symmetry (space group No. 62) and lattice parameters *a* = 5.51 Å, *b* = 10.14 Å, and *c* = 4.69 Å. Small discrepancies in the relative intensities between the measured patterns and the reference were related to the presence of the Tb and Tm doping. They could also be caused by the creation of structural defects, e.g., antisite defects, whose appearance is common in the case of melt-growth techniques, as well as by the preferred grain orientation induced by the sample preparation procedure.

### 3.2. Intrinsic Defect-Related Luminescence

**Figure 3:**
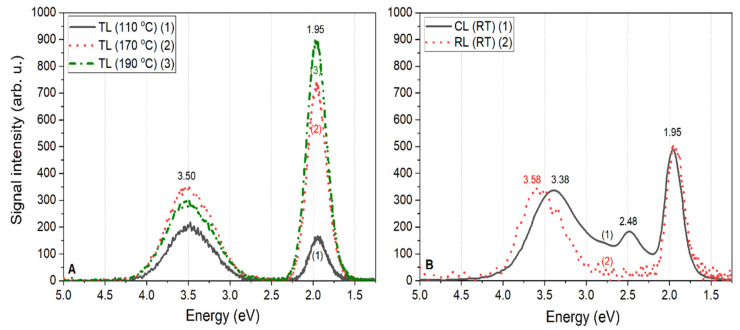
Intrinsic defect-related luminescence spectra measured for nominally undoped LiMgPO_4_ crystals under the different modes of stimulation: thermoluminescence (TL) (**A**), and cathodoluminescence (CL) and radioluminescence (RL) (**B**). One should note that the CL and RL spectra were normalized, and thus their intensities should not be directly compared to the intensities of the TL spectra.

The intrinsic defect-related luminescence spectra measured for nominally undoped LiMgPO_4_ crystals are shown in Figure 3A,B. The TL spectra registered at different temperatures (110 °C, 170 °C, and 190 °C, corresponding to the TL peak maxima), shown in Figure 3A, were compared with the cathodo- and radioluminescence spectra measured at room temperature, shown in Figure 3B. It should be noted here that the intensities of the CL and RL spectra were normalized, and thus should not be directly compared to the TL spectra intensities. The TL spectra were not normalized, and thus present the real behavior due to the increasing temperature of the sample. It can be seen that the measured spectra consisted, in most cases, of two broad bands located at around 3.50 and 1.95 eV, corresponding to 354 and 635 nm, respectively. The intensity of the UV emission band (354 nm) increased with temperature up to around 170 °C and then started decreasing. For temperatures higher than around 300 °C, this band was practically invisible and the whole measured luminescence originated from the second band (635 nm) [19]. The shape of the spectra did not change with increasing temperature and agreed very well with the RL spectrum measured at RT. The CL spectrum measured at RT showed a slightly different shape. The 1.95 eV emission band fitted very well with both the TL and RL emission spectra; however, the UV emission was shifted a little toward lower energies. The maximum of this CL emission band peaked at around 3.38 eV. Moreover, an additional band with a maximum at around 2.48 eV could also be seen in the CL spectra. This was an exceptional case since this middle-range 2.48 eV emission band could not be observed for either of the TL nor RL emission spectra. It is not presently clear how the 10 keV electron beam interaction with the thin surface layer (of around 1 µm) of the sample could produce an additional peak at 2.48 eV (see Figure 3B). The proposed explanation is connected to the presence of a larger concentration of surface defects and/or their aggregates with incidental impurities.

According to available literature data, one can try to explain the origin of the observed emission spectra. The broad emission band peaks at around 3.50, 2.48, and 1.95 eV were probably related to the luminescence of F^+^ and F centers, and possibly their aggregates. Namely, a 3.50 eV band could correspond to the luminescence of F^+^ centers. It is commonly known that the F-like centers tend to aggregate in more complex structures, e.g., F_2_ and F_3_^+^ centers, which were probably responsible for the observed emissions at 2.48 and 1.95 eV. Meanwhile, the origin of the middle-range 2.48 eV emission is not easy to explain as this emission was observed only in the case of the CL spectrum. This may have been related to completely different measurement conditions since the low-energy (10 keV) electron beam only interacted with the surface layer of the sample, which was approximately 1 µm thick, and the only emission from this layer was recorded. Moreover, the whole measurement was conducted at RT. In the case of the TL measurements, the signal originating from the whole sample volume was registered for temperature increases from RT even up to 500 °C. Therefore, this could have influenced the observed differences. In the case of the RL measurements, the signal registration also took place at RT, as with the CL case, but the radiation from an external beta-irradiator excited the whole sample uniformly, in contrast to the CL measurements, where only the sample surface (usually with a large concentration of defects) was stimulated. Therefore, the presence of any surface impurities may have influenced the measured CL spectra.

### 3.3. PL Spectra and Decay Kinetics Analysis for the Undoped LMP Crystal

**Figure 4:**
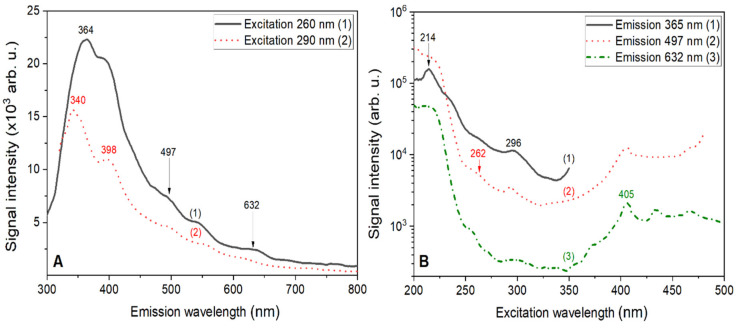
Photoluminescence (PL) emission (**A**) and excitation (**B**) spectra measured for the undoped LiMgPO_4_ crystal investigated within this work. The excitation wavelengths in (**A**) (260 and 290 nm) corresponded to the maxima in the excitation spectra in (**B**). The emission channels in (**B**) corresponded to the emission maxima observed using TL, CL, and RL measurements in the spectra shown in Figure 3.

The emission spectra measured for the undoped LMP crystal under the excitation wavelengths of 260 and 290 nm are shown in Figure 4A. The spectra exhibited complicated shapes with at least several strongly overlapping peaks, which suggests the presence of different types of emission centers related to intrinsic structural defects in the LMP host. The PL spectra (Figure 4A) extended over the wavelengths of both the UV and visible ranges up to around 700 nm, but the UV emission bands peaks at 340, 364, and 398 nm were dominant. The intensity of the excitation spectrum decreased with increasing excitation wavelength, but the shape of the spectrum remained unchanged. This confirmed the correspondence of 260 and 290 nm excitation bands to the same emission centers. It should be noted here that the PL spectra obtained in this work were in good agreement with results presented in Kellerman et al. [20] for cold-pressed and sintered LMP pellets regarding both the shape of the spectra and the observed characteristics.

The PL excitation spectra recorded at emission channels corresponding to the maxima of emission shown in Figure 3 are presented in Figure 4B. Since the LiMgPO_4_ compound is a relatively wide band-gap material (E_g_ = 5.5 eV), the excitation peaks can be related to (I) the transitions of the charged carriers from the valence band to the defect-related states within the band-gap; (II) the transitions from the excited states of the defects to the conduction band; and (III) intrinsic transitions of the defect states, namely F^+^ and F centers, as well as their aggregates. All excitation spectra measured at RT showed an intense band with a long-wavelength edge at around 214 nm. This value agrees very well with the long-wavelength edge of fundamental absorption [20]. The excitation spectra, measured at emission wavelengths of 365, 497, and 632 nm, exhibited weak excitation bands at around 260 and 295 nm. Meanwhile, these excitation bands were strongly reduced in the spectrum measured at 632 nm, but another band peak at around 405 nm became dominant. This small intensity of the 260 and 295 nm bands, compared to the high-energy band (225 nm), suggests that transitions responsible for these bands could correspond to the intrinsic transitions of the defect states inside the forbidden band. Moreover, taking into account the fact that the probability of such transitions is much lower than the probability of charge transfer transitions from the valence to the conduction band, such an explanation seems to be justified.

**Figure 5:**
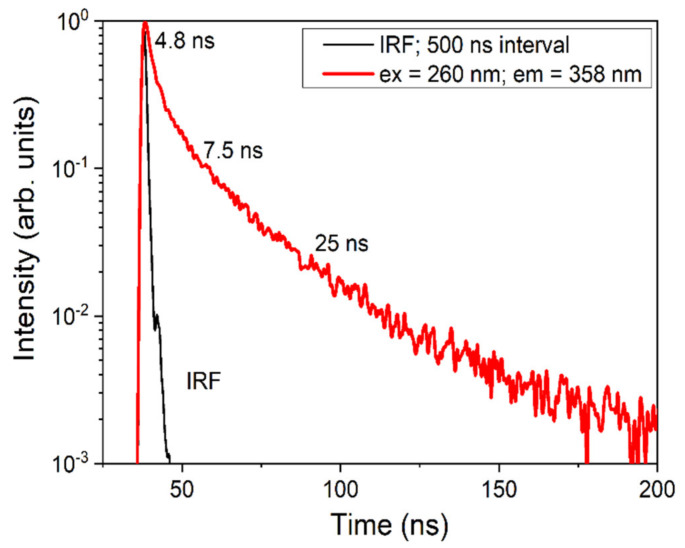
Decay kinetics of the UV luminescence for the undoped LiMgPO_4_ crystal under excitation at 260 nm in the vicinity of the respective excitation band. The instrumental response function (IRF) is also presented.

The decay kinetics of UV luminescence measured for the undoped LMP crystal sample at 358 nm, under the excitation at 260 nm, is shown in Figure 5. This decay curve shows a fast, non-exponential decay as a superposition of three components with decay times of 4.8, 7.5, and 25 ns, probably corresponding to the different emission centers. That is, the faster decay component of the luminescence with a decay time of 4.8 ns could correspond to the F^+^ centers’ luminescence when other fast decay components may be assigned to the F^+^-like aggregate centers. The data of the decay kinetics of F^+^ centers in other oxide compounds, e.g., Al_2_O_3_, YAlO_3_, or Y_3_Al_5_O_12_ [26,27,28], may constitute a reliable confirmation of the conclusions drawn for the LMP compound from this study’s investigations.

### 3.4. RE Dopant-Related Luminescence

**Figure 6:**
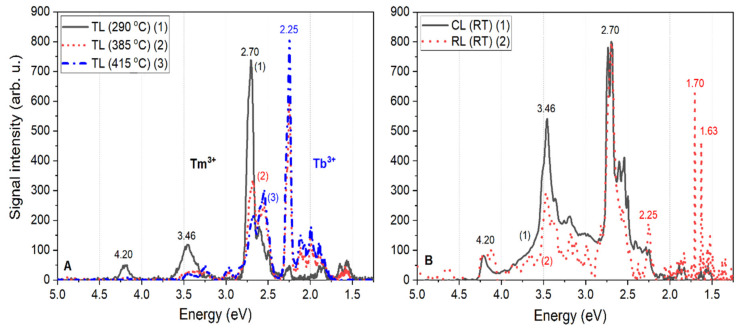
Luminescence spectra measured for double-doped LiMgPO_4_:Tb,Tm crystals under different modes of stimulation: TL (**A**) and CL and RL (**B**). Note that the CL and RL emission spectra were normalized, and thus their intensities should not be directly compared to the intensities of the TL emission spectra.

The luminescence emission spectra related to the Tb^3+^ and Tm^3+^ dopants introduced to the LMP matrix are shown in Figure 6A,B, where the most intense transitions are labeled. The TL spectra measured at different temperatures (290 °C, 385 °C, and 415 °C), shown in Figure 6A, were compared with the CL and RL spectra measured at RT, presented in Figure 6B. It should be noted here that the intensities of the CL and RL spectra were normalized, just as in Figure 3, and thus should not be directly compared to the TL spectra intensities. The TL spectra were not normalized, and thus represent the real behavior due to the increasing temperature of the sample during the measurements.

Regarding the TL emission spectra, it is remarkable that in the low-temperature range, up to around 290 °C, the observed blue emission that mainly originated from Tm^3+^-related centers, are the characteristic transitions visible in Figure 6A. These transitions at 4.20, 3.46, and 2.70 eV (295, 355, and 460 nm, respectively) corresponded to the ^1^I_6_→^3^H_6_, ^1^D_2_→^3^H_6_, and ^1^G_4_→^3^H_6_ transitions of the Tm^3+^ ions, respectively. Meanwhile, we also note here that the band observed at around 355 nm probably resulted from a superposition of the ^1^I_6_→^3^F_4_ and ^1^D_2_→^3^H_6_ transitions, which could not be better resolved because of the low resolution (4 nm) of the applied spectrometer. Other less-intense transitions ascribed as characteristic for Tm^3+^ ions [19] were also observed. The emissions of the Tb^3+^ ions were not practically visible at this temperature range. Only a very low-intensity peak at 2.25 eV, which probably came from the ^5^D_4_→^7^F_5_ transition of Tb^3+^, was visible in the emission spectrum below 290 °C. The situation changed dramatically when the temperature increased. At a temperature around 385 °C, the ^1^I_6_→^3^H_6_, ^1^I_6_→^3^F_4_ and ^1^D_2_→^3^H_6_ transitions of Tm^3+^ practically disappeared; the ^1^D_2_→^3^F_4_ transition decreased significantly; and the characteristic transitions of Tb^3+^ ions became visible, namely the characteristic group of bands located around 2 eV, corresponding to ^5^D_4_→^7^F_4,3,2_ transitions. This characteristic green Tb^3+^ emission increased with temperature such that at around 415 °C, the emissions from Tm^3+^ ions practically stopped and the emissions of Tb^3+^ ions (mostly from ^5^D_4_ level) were the only ones visible. Such a temperature dependence of emission suggested an unusual recombination mechanism, the model of which is proposed later in the paper. In the case of the CL and RL spectra measured at RT (Figure 6B), mainly the emission from Tm^3+^ was observed, similar to the observation from the TL emission spectra in the low-temperature range. These differences could be related to the temperature at which the measurements were performed and may also indicate that the Tb^3+^ emission centers in double-doped LMP crystals became active at higher temperatures. It is also worth noting that in the case of the RL emission spectra, transitions at 1.70 and 1.63 eV (corresponding to the ^1^D_2_→^3^F_2,3_ transitions of Tm^3+^, respectively) were probably observed, which could not be registered using measurements with the other modes of excitation, namely TL and CL. However, the overall signal intensity in the case of RL measurements was not high enough to draw reliable conclusions.

### 3.5. PL Spectra and Decay Kinetics Analysis for Double-Doped LMP Crystals

**Figure 7:**
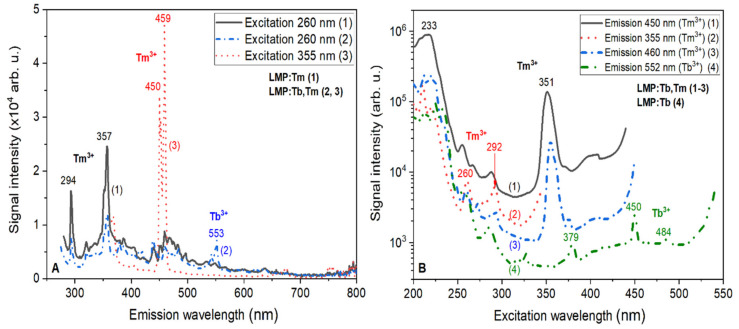
Emission (**A**) and excitation (**B**) spectra measured for LiMgPO_4_:Tm and LiMgPO_4_:Tb,Tm crystals investigated within this work. The emission spectra shown in (**A**) were registered under excitation in the respective excitation bands of Tm^3+^ ions visible in (**B**). The excitation spectra shown on (**B**) were registered in the emission maxima corresponding to the luminescence of Tm^3+^ ions (355 nm and 450–460 nm) and Tb^3+^ ions (550 nm).

The PL emission spectra of LiMgPO_4_:Tb,Tm crystals (Figure 7A) under excitation in the Tm^3+^-related excitation bands at 260 and 355 nm (Figure 7B) showed the dominant luminescence of Tm^3+^ ions of the main band peaks at 297, 357, and 450–457 nm, as well as the low-intensity emission bands of Tb^3+^ ions in the main peak at 553 nm. Generally, such PL emission spectra (Figure 7A) correlated with CL and RL spectra of the LiMgPO_4_:Tb,Tm crystals shown in Figure 6B. The excitation spectra of the Tm^3+^ luminescence in the different emission bands (see Figure 7B, curves 1–3) showed sets of bands with maxima at 351, 296, and 260 nm, corresponding to the respective 4f–4f transitions of Tm^3+^ ions. The positions of these excitation bands correlated with the respective Tm^3+^ emission bands (Figure 6A). The excitation spectrum of the Tb^3+^ luminescence presented the low-intensity band peaks at 379, 450, and 484 nm, corresponding to transitions from the excited ^5^D_3_ level of Tb^3+^ ions, as well as several bands corresponding to the Tm^3+^ transition at 260 nm. Therefore, we can infer the excitation of Tb^3+^ luminescence via the emissions from Tm^3+^.

**Figure 8:**
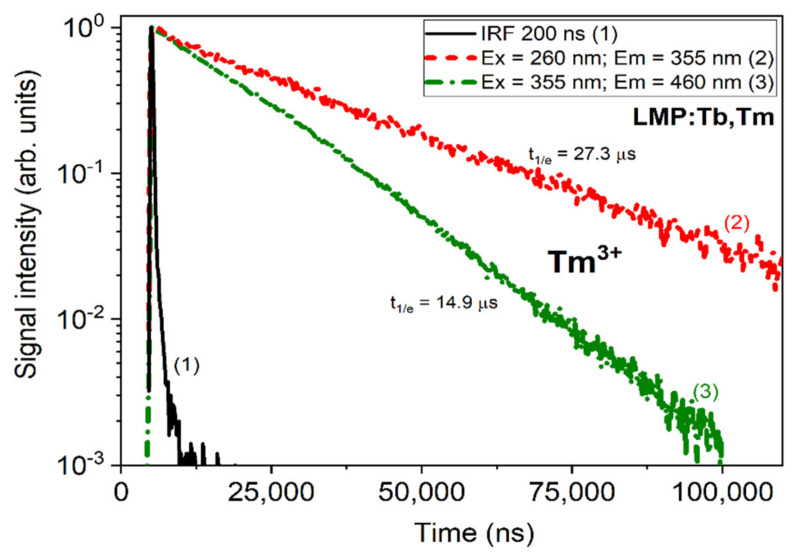
The luminescence decay kinetics of Tm^3+^ ions at 355 nm and 460 nm under excitation in the respective excitation bands of Tm^3+^ ions. The instrumental response function (IRF) is also presented.

**Figure 9:**
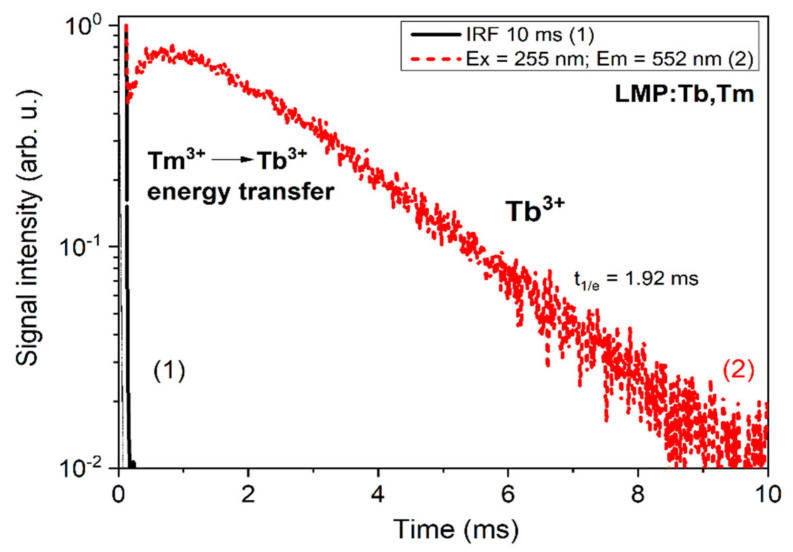
The luminescence decay kinetics of Tb^3+^ ions at 552 nm under excitation in the respective excitation band of Tm^3+^ ions. The instrumental response function (IRF) is also presented.

Figure 8 shows the decay kinetics of the Tm^3+^ ions’ luminescence in the main emission band peaks at 355 and 460 nm under excitation in the bands at 260 and 355 nm, respectively. The respective decay times were equal to 14.9 and 27.3 µs. The decay kinetics of the Tb^3+^ ions’ luminescence under excitation in the band at 260 nm, corresponding to both Tb^3+^ and Tm^3+^ ions, displayed a complicated form with a typical initial rise-time part of decay curve (Figure 9). Such a rise-time part of the decay curve indicated the presence of the energy transfer between Tm^3+^ and Tb^3+^ ions. Furthermore, the decay kinetics of the Tb^3+^ ions’ luminescence in the LiMgPO_4_ host with a decay time of 1.92 ms (Figure 9) was much slower in comparison with the Tm^3+^ luminescence (Figure 8).

It is remarkable that in the case of thermal stimulation (see Figure 6A), the emission was mainly from Tm^3+^, while the Tb^3+^ emission centers only became active at temperatures above 290 °C. In the case of the optical excitation at RT (see Figure 7A), the emission was also mostly from Tm^3+^. Such behavior of the TL and PL measurements could be related to the opposite trapping nature of the studied RE ions, namely to create the hole and electron trapping sites, respectively, as it was predicted by a semi-empirical model of Dorenbos [22]. The other possible explanation suggests the differences in the applied measurement techniques (modes of stimulation/excitation) are the cause.

### 3.6. A Simple Model of TL in Double-Doped LMP Crystals

First of all, recall that the spectra measured for the single-doped (with Tb or Tm) LMP crystals revealed the emission bands that are characteristic for both doping ions. In the case of the Tb-doped samples, transitions from both the ^5^D_4_ and ^5^D_3_ levels were observed over the whole temperature range. Similarly, in the case of the Tm-doped crystals, the transitions from the ^1^D_2_ level were observed over the whole temperature range [19]. The situation changed significantly for the Tb plus Tm double-doped LMP crystals. Here, in the low-temperature range, only the characteristic emission from the ^1^D_2_-related level of Tm^3+^ ions was observed (see Figure 6A). When the temperature rose, the Tm^3+^ emission gradually decreased and the transitions from the ^5^D_4_-related level of Tb^3+^ started to be visible. Above 400 °C, the emissions of Tm^3+^ ions were practically invisible and the Tb^3+^ emission became dominant.

**Figure 10:**
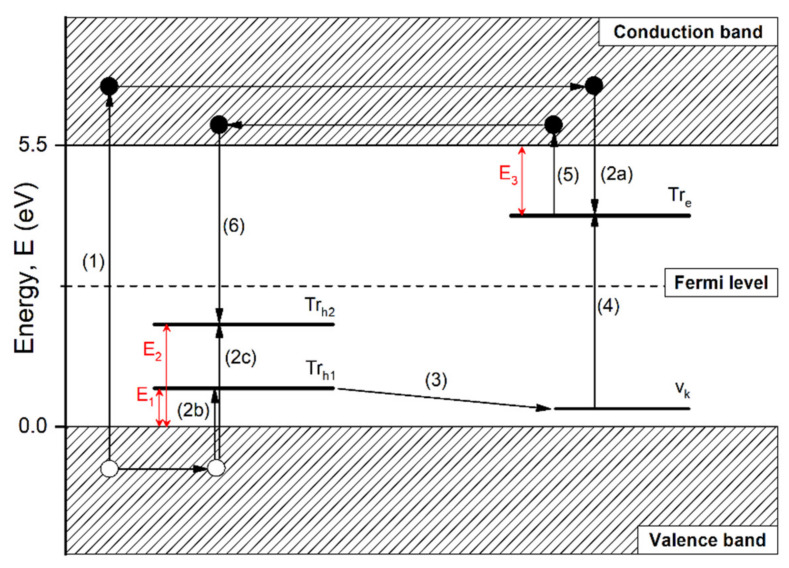
A simplified model of thermoluminescence observed in double-doped LiMgPO_4_:Tb,Tm crystals. The arrows indicate consecutive processes involved in the TL phenomenon. These are as follows: (1) the creation of free charge carriers by ionizing radiation, (2) the trapping of charge carriers by Tm-related electron trapping sites (2a), the Tb-related hole trapping sites (2b, 2c), (3) the hole release and creation of a V_k_-center, (4) the recombination of a V_k_ center with the electron trapped in *Tr_e_*, (5) electron release to the conduction band, (6) recombination of the electron with the hole trapped in the Tb-related trapping site.

Based on these observations, a simplified model of thermoluminescence mechanisms in double-doped LiMgPO_4_ crystals was proposed and is shown in Figure 10. The model assumed the existence of two Tb^3+^-related hole trapping levels denoted as *Tr_h1_* and *Tr_h2_*, which correspond to the ^5^D_3_ and ^5^D_4_ energy levels of the Tb^3+^ ions, respectively. The *Tr_e_* level denotes the Tm^3+^-related electrons’ trapping level. The energies of these three trapping levels are denoted as *E_1_*, *E_2_*, and *E_3_*, respectively, and the observed TL properties suggest that E_1_ < E_3_ < E_2_. V_k_ denotes the energy level of the V_k_ center (a hole that is shared between two neighboring oxygen anions in the host lattice), which is located 0.7–1.0 eV above the top of the valence band in oxide compounds [29]. Taking into account the above-mentioned assumptions, one can explain the TL emission observed for the studied double-doped LMP crystal samples. The first stage of the TL phenomenon, namely the creation and trapping of free charge carriers, will be omitted since one can assume that the samples were successfully irradiated, thus free charge carriers were created and trapped in the appropriate trapping levels. These processes are denoted in Figure 10 by the arrows marked (1) and (2a, 2b, and 2c). Therefore, only the second stage of the TL phenomenon related to the thermal stimulation of the sample is discussed.

Recently, a similar double-doped YPO_4_:Tb,Tm yttrium orthophosphate was investigated in Bos et al. [29]. Based on the observed TL properties, the authors concluded that Tb^3+^-related hole trapping sites and Tm^3+^-related electron trapping sites are energetically similar (only 0.2 eV difference). This suggested that both kinds of charge carriers become mobile in a similar temperature range, giving rise to simultaneous emission from both Tb- and Tm-related levels. In the case of LiMgPO_4_ crystals, the situation looks somewhat different. First of all, it seems that the energy difference between Tb- and Tm-related trapping levels was significant, given that up to 290 °C, only the emission from Tm^3+^ was observed (see Figure 6A). This suggests that with increasing temperature, the holes were released first from the Tb-related trapping levels (arrow (3) in Figure 10) and recombined with the electrons trapped in the Tm-related levels (arrow (4) in Figure 10), giving rise to the characteristic emission of Tm^3+^ ions. However, this recombination probably did not occur through the top of the valence band, but via the V_k_-center level, as was suggested and explained in Bos et al. [29]. When the temperature was appropriately high (above 300 °C), the electrons occupying the Tm-related trapping sites started to be released into the conduction band (arrow (5) in Figure 10), where they migrated and recombined with holes trapped in the ^5^D_4_-related Tb^3+^ sites, giving rise to the characteristic emission of Tb^3+^ ions. Simultaneously, the population of holes occupying the *Tr_h1_* level (which is probably related to the ^5^D_3_ level of Tb^3+^) in Figure 10 significantly diminished, as seen by the observed luminescence emission from Tm^3+^ decreasing quite fast, and at the highest temperatures, the emission from the ^5^D_4_ level of Tb^3+^ became dominant (Figure 6A). It is worth mentioning that the emission from the ^5^D_3_ level of Tb^3+^ was practically invisible. This implies that the *Tr_h1_* and *Tr_h2_* levels in Figure 10 were probably related to the ^5^D_3_ and ^5^D_4_ levels of Tb^3+^, respectively. This simple model explains the temperature dependence observed for double-doped LiMgPO_4_ crystals luminescence presented in Figure 6A and may be fully applicable to the emission of other double-doped compounds.

## 4. Conclusions

In this work, the luminescence properties of undoped, Tm^3+^ doped, and Tb^3+^ plus Tm^3+^ double-doped lithium magnesium phosphate crystals were investigated. An intrinsic and dopant-related luminescence was studied using cathodo-, radio-, photo-, and thermoluminescence methods. Double-doping with Tb^3+^ and Tm^3+^ ions was analyzed as these dopants were expected to exhibit an opposite trapping nature, namely to create the hole and electron trapping sites, respectively.

The spectra measured for the undoped samples revealed three prominent broad emission bands with maxima at around 3.50, 2.48, and 1.95 eV. The first one probably originated from excitons localized around the oxygen vacancies and the luminescence of F^+^ and F centers. The more complex aggregates of F centers, namely F_2_ and F_3_^+^ centers, were probably responsible for a 1.95 eV emission band. The luminescence spectra measured for Tb^3+^ plus Tm^3+^ double-doped crystals showed characteristic peaks corresponding to the 4f–4f transitions of the studied RE dopants.

A simplified model of the TL recombination mechanism was proposed to explain the observed temperature dependence of the luminescence emission. It seems that in the low-temperature range, the charge carriers were released from the ^5^D_3_-related Tb^3+^ trapping sites and recombined in the Tm-related sites, giving rise to the characteristic Tm^3+^ emission. At higher temperatures, above 300 °C, the electrons in the Tm^3+^-related trapping sites started to be released and recombine at the ^5^D_4_-related Tb^3+^ recombination centers, giving rise to the characteristic emission of Tb^3+^. This model explains the temperature dependence observed for double-doped LiMgPO_4_ crystals’ luminescence emission and may be fully applicable to the emission of other double-doped compounds.
